# Assessment of knowledge, attitudes, practices, and self-efficacy toward evidence-based medicine among resident physicians: a cross-sectional study from Qatar

**DOI:** 10.3389/fmed.2025.1651632

**Published:** 2025-09-23

**Authors:** Wafa Mohammed Ahmed, Mohamed Aabdien, Abdelaziz Mohamed, Mohamed Iheb Bougmiza

**Affiliations:** ^1^Community Medicine, Hamad Medical Corporation, Doha, Qatar; ^2^Community & Preventive Medicine, Primary Health Care Corporation, Doha, Qatar; ^3^Internal Medicine, Hamad Medical Corporation, Doha, Qatar; ^4^Community Medicine Residency Program, Primary Health Care Corporation, Doha, Qatar

**Keywords:** residents, residency training, self-efficacy, practice, attitude, knowledge, evidence-based medicine

## Abstract

**Introduction:**

Evidence-based medicine (EBM) is essential for delivering high-quality healthcare. However, no studies have yet assessed the practice of evidence-based medicine among resident doctors in Qatar. This study aimed to examine the key factors influencing EBM practice, focusing on the physicians’ self-efficacy, self-reported knowledge, and their attitudes toward EBM.

**Methodology:**

An analytical cross-sectional study design was used, using a total population sampling method. Data were gathered through a validated questionnaire to assess EBM practices among resident physicians at HMC. Descriptive statistics, bivariate analysis, and multivariate analysis were used to analyze the data.

**Results:**

A total of 355 resident physicians participated in the survey. The average age of the participants was 28.3 years. The overall EBM practice score averaged 38, indicating a moderate level of practice according to the Bloom scale. Significant associations were found between EBM practice and physician age, gender, work experience, and prior EBM training. The majority of participants demonstrated moderate knowledge of EBM but had low attitudes toward its application. Additionally, the resident physicians exhibited low self-efficacy in applying EBM.

**Conclusion:**

While the work environment in Qatar is highly supportive of EBM practice and the resident physicians possess strong academic qualifications, their overall practice of EBM remains moderate. This could be attributed to their relatively young age, limited experience, and unfavorable attitudes toward EBM, along with low self-efficacy. There is a need for targeted training workshops to improve EBM skills and attitudes among resident physicians, which could enhance their practice and, ultimately, improve patient outcomes in Qatar.

## 1 Introduction

Competent clinical decision-making is a multifaceted and essential aspect of modern medical care (1). It necessitates the integration of evidence-based practice (EBP), which is a foundational element of high-quality, patient-centered healthcare (2). Given the continuous expansion of medical knowledge, evidence-based medicine (EBM) plays a vital role in keeping healthcare providers informed. It supports clinical decisions by providing access to the most current research, clinical guidelines, and evidence (3).

The concept of EBM originated at McMaster University in the 1980s (4), and it was later endorsed by several educational institutions, including the Accreditation Council for Graduate Medical Education (ACGME), which designated it as a core element of practice-based learning and improvement (5). Despite the widespread recognition of EBP principles, their consistent application in clinical practice remains limited among many physicians (6).

Systematic reviews (7–9) have identified a gap between the production of scientific knowledge and its practical use in healthcare settings. These reviews also reveal multiple obstacles, including factors related to patients, professionals, organizational environments, and healthcare systems, which hinder the implementation of EBP.

The adoption of EBM by the ACGME (5) has motivated residency programs to integrate EBP into their educational frameworks (10). Numerous studies have explored healthcare providers’ knowledge, attitudes, and practices (KAP) regarding EBM at both regional and international levels (11–14). One study, for instance, assessed EBM among resident physicians in Syria and reported low awareness, a neutral attitude, and poor practice (15).

Healthcare providers in Qatar come from diverse medical backgrounds, and the country’s health system is rapidly evolving. Understanding how evidence-based practice (EBP) is perceived and implemented by medical residents is essential. It helps identify areas where educational interventions are needed and guides efforts to better integrate research findings into clinical care. Additionally, it highlights key areas that may require strategic investment to improve clinical outcomes (16). To our knowledge, no study has yet evaluated EBM practice among junior physicians in Qatar. Only one related study, conducted in 2010, focused on primary care physicians in the country (17). Therefore, this study aims to assess evidence-based healthcare practice among residents in Qatar’s teaching hospitals by analyzing their self-efficacy, knowledge, and attitudes toward EBM to identify educational gaps and support its wider adoption.

## 2 Materials and methods

### 2.1 Study design and study setting

A cross-sectional study was conducted between April and June 2024 to evaluate evidence-based medicine (EBM) practices among resident physicians in Qatar. The study targeted residents from all medical specialties—both surgical and non-surgical—who were working at Hamad Medical Corporation (HMC). All hospitals under HMC that offer residency training programs, as regulated by HMC’s Medical Education Department (the national authority responsible for residency training in Qatar), were included.

### 2.2 Study procedure

The target population included male and female residents currently enrolled in residency programs at HMC. Those excluded from the study included individuals who were unable to provide informed consent, as well as interns and fellows not classified as resident doctors.

The Institutional Review Board of Hamad Medical Corporation issued approval (Ref no.: MRC-01-23-534). All procedures in this study were performed in accordance with the Declaration of Helsinki. Participants were invited to participate voluntarily and were provided with comprehensive information about the study. Informed consent was implied by the participants’ completion of the questionnaire.

#### 2.2.1 Sample size and sampling method

The required sample size for this study was estimated using the Raosoft sample size calculator (18). Considering a total of approximately 645 resident physicians enrolled in Hamad Medical Corporation (HMC) residency programs, a minimum of 218 respondents was determined to be adequate. This estimation was based on a 95% confidence interval, a 5% margin of error, and a presumed response distribution of 50%. All residents who met the eligibility criteria for HMC Medical Education were invited to participate in the research.

#### 2.2.2 Measurement tools and data collection process

Although multiple validated instruments exist to evaluate EBM, such as the Fresno, Berlin, Baum, McColl, and EBM Questionnaire (19–27), we chose the Noor Evidence-Based Medicine Questionnaire (28). The Noor tool was selected to evaluate knowledge, attitudes, and practices (KAP) related to EBM due to its recent development and validation in a younger demographic that closely resembles our study population. Its strong psychometric properties and relevance to our research objectives made it a suitable choice.

The survey was structured into six distinct sections. The first section gathered demographic and professional background data, including variables such as age, gender, postgraduate year (PGY) level, nationality, marital status, medical specialty, years since graduation, highest academic degree, current workplace, average number of patients seen daily, and the availability of Internet access, online databases, quick reference applications, and continuing medical education at the workplace.

Sections 3–5 were derived from the validated Noor EBM Questionnaire, which includes 15 items on knowledge, 17 on attitudes, and 11 on practices toward EBM (28). Responses to knowledge and attitude questions were recorded using a 5-point Likert scale ranging from “Strongly Disagree” (1) to “Strongly Agree” (5). Practice items were rated on a frequency scale from “Never” (1) to “Always” (5). Raw scores were computed for each domain and subsequently converted into percentage scores. These were then classified using Bloom’s cutoff criteria (29): scores below 59% indicated low/poor levels, scores between 60% and 80% reflected moderate or fair levels, and scores above 81% represented high/good levels of knowledge, attitude, or practice (30).

Section 6 evaluated participants’ self-efficacy in applying EBM using only the self-efficacy domain of the I-SABE instrument, a validated and reliable tool specifically designed to measure confidence in practicing evidence-based healthcare (31). A 75th percentile cutoff was applied to differentiate between high and low levels of self-efficacy, consistent with previous studies (32, 33).

The questionnaire was administered exclusively in English, as all participating healthcare professionals had an advanced level of English proficiency. It consisted of 64 items and required approximately 25 min to complete. The questionnaires used in this study were previously published and are openly accessible. Authors of the tools permit their use non-commercially (28, 31).

Prior to the main data collection phase, a pilot test involving 10 resident doctors was conducted to assess the clarity, relevance, and timing of the questionnaire. Feedback from the pilot was used to confirm the comprehensibility and acceptability of the tool, though the pilot data were not included in the final analysis. The finalized questionnaires were distributed manually through in-person visits to resident doctors. For those who did not respond initially, two follow-up visits were made to encourage participation.

The questionnaires used in this study were previously published and are openly accessible. The authors permit their non-commercial use.

### 2.3 Statistical analysis

Data were analyzed using IBM SPSS Statistics for Windows, version 26 (IBM Corp., Armonk, NY, USA). Descriptive statistics, including means, standard deviations, frequencies, and percentages, were used to summarize the dataset and were displayed in relevant tables and figures. EBM practice score normality was assessed using the mean and standard deviation, the shape of the normal distribution curve, and the Kolmogorov–Smirnov test. To examine associations between variables, independent sample *t*-tests and one-way ANOVA were applied where appropriate. Furthermore, multivariate analysis was performed using multiple linear regression to explore the relationship between the key outcome variable and independent predictors.

## 3 Results

**Figure 1:**
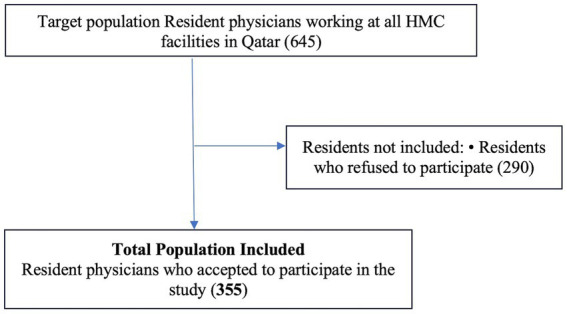
Flow chart of recruitment of study participants. The data have been presented as numbers *N*.

At the time of data collection, a total of 645 residents were registered under HMC. Of these, 355 agreed to take part in the study (see Figure 1). The most commonly cited reason for non-participation among those who declined was lack of time due to busy schedules.

### 3.1 Demographics and factors associated with EBM practice among medical residents

The average age of participants in this study was 28.3 years (±3.03). Of the total respondents, males represented a slight majority, accounting for 180 individuals (50.6%). In terms of nationality, the majority were non-Qatari (298, 84.2%), while Qatari nationals comprised 56 (15.8%) of the sample (Table 1).

**Table 1:** Demographics and factors associated with EBM practice of the medical resident participants (*N* = 355).

Variable	Frequency	Percentage	Mean	SD
Age	–	–	28.3	3.03
24–29	181	70.7%	–	–
30–40	75	29.3%	–	–
Gender				
Male	180	50.7%	-	–
Female	170	47.9%	–	–
Non-response	5 (1.4%)	1.4%	–	–
Marital status				
Married	207	58.2%	–	–
Single	139	39.3%	–	–
Divorced	4	1.1%	–	–
No response	5	1.4%	–	–
Residency program				
Internal medicine	103	29.1%	–	–
Other specialties	252	79.9%	–	–
Time since graduation	4.47	2.56	–	–
Work experience	2.78	1.968	–	–
Nationality				
Qatari	56	15.8%	–	–
Non-Qatari	299	84.2%	–	–
Years of residency				
Junior	213	60%	–	–
Senior	142	40.0%	–	–
Previous training in EBM				
Yes	152	42.7%	–	–
No	179	55.3%	–	–
No response	6	1.7%	–	–
Previous research experience				
No	32	9%	–	–
Yes	316	89%	–	–
No response	7	2.0%	–	–
Internet access at the workplace				
Yes	340	95.8%	–	–
No	8	2.3%	–	–
No response	7	2%	–	–
Access to databases at the workplace				
Yes	315	88.7%	–	–
No	32	9%	–	–
No response	8	2.3%	–	–
Medical education				
Yes	342	96.3%	–	–
No	6	1.7%	–	–
No response	7	2%	–	–

The data have been presented as *N*, %, and mean ± SD.

With respect to medical specialty, internal medicine residents made up approximately one-third of the participants (103 residents; 29.1%), while the remaining two-thirds represented various other specialties. The average duration of clinical practice among participants was 2.7 years (± 1.9). The majority of respondents were in their 1st, 2nd, or 3rd year of residency training (213; 60%), and the average time since graduation from medical school was 4.5 years (Table 1).

**Figure 2:**
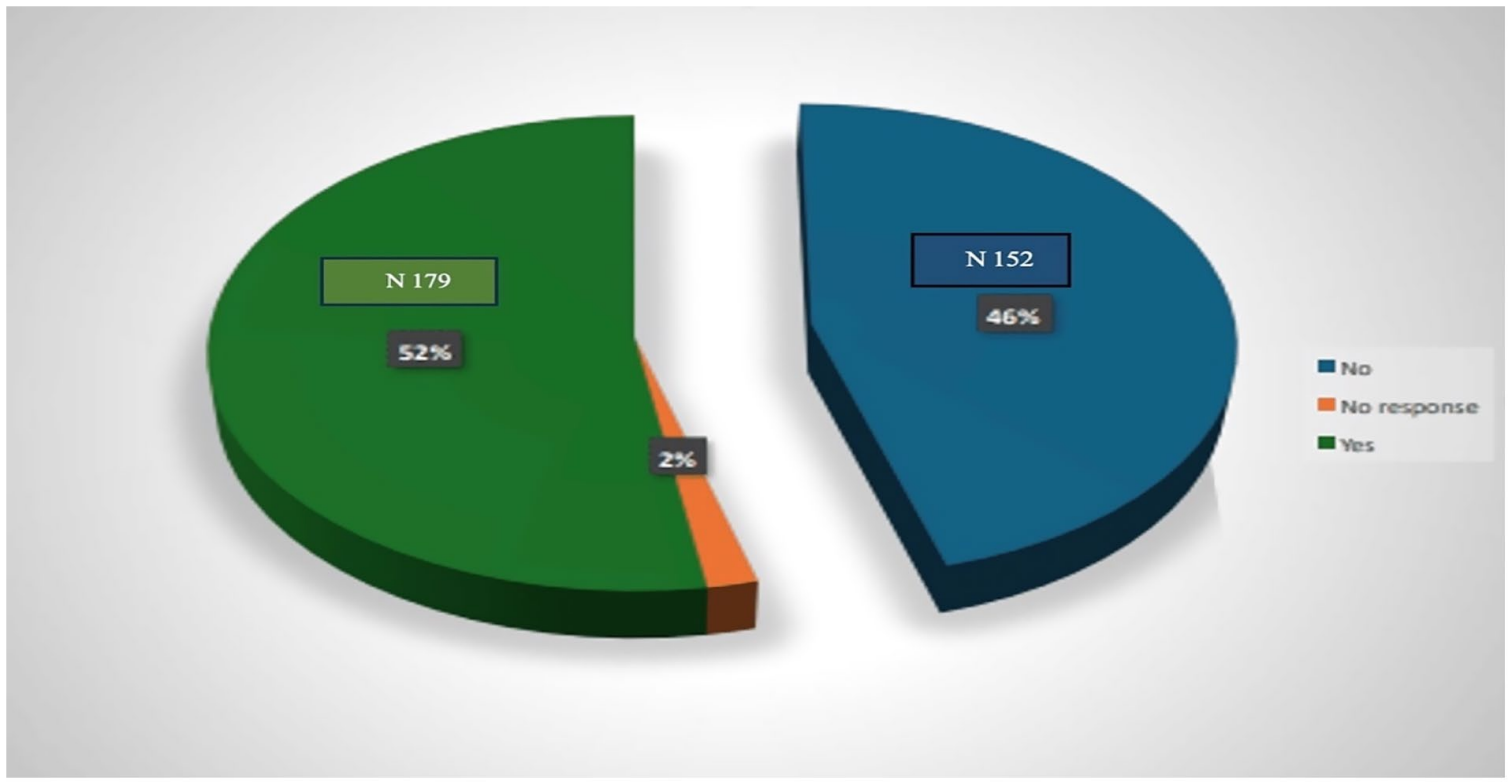
Percentage of resident physicians who had previous training in EBM at HMC in Qatar 2024 (*N* = 355). The data have been presented as *N* and percentages %.

Regarding familiarity with evidence-based medicine (EBM), most residents reported prior exposure to EBM training, primarily through face-to-face sessions, as illustrated in Figure 2. Additionally, approximately 90% of the participants had previous research experience. The working environment at HMC was generally viewed as supportive of EBM practice. Nearly all residents reported consistent access to Internet services at their workplace, the availability of institutional subscriptions to online medical databases, and frequent opportunities for continuing medical education to keep their clinical knowledge and skills current.

### 3.2 Resident physicians’ EBM domain scores

The results from Table 2 describe resident physicians’ scores in knowledge, attitudes, and practices of evidence-based medicine. Total scores were calculated for each domain. Each raw score was converted into a percentage score and categorized based on Bloom’s cutoff criteria. Scores under 59% were interpreted as a low level of practice, inadequate knowledge, or poor attitude. Scores between 60% and 80% were considered moderate, neutral, or fair. Scores above 81% reflected high knowledge, positive attitude, or good practice.

**Table 2:** Scores of resident physicians’ knowledge, attitude, and practice of evidence-based medicine at Hamad Medical Corporation (*N* = 355).

EBM scale	Mean	Median	SD	IQR	Skewedness coefficient	Kurtosis coefficient	Mode	Range	Bloom low	Bloom moderate	Bloom high
Knowledge domain	51.69	51.00	6.09	48–55	−0.709	8.414	48.00	8–73	17/4.8%	296/83.4%	42/11.8%
Attitude domain	67.24	68.00	7.53	61–73	−0.008	−0.642	64.00	48–85	203/57.2%	130/36.6%	22/6.2
Practice domain	38.49	39.00	6.33	35–42	−0.188	−0.228	40.00	18–55	49/13.8%	221/62.3%	85/23.9
Self-efficacy domain	28.64	29.00	3.68	27–31	−0.705	1.278	30.00	13–35	289/81.4%		66/18.6%

The data have been presented as mean, median, SD, IQR, mode, range, and loom classification.

**Figure 3:**
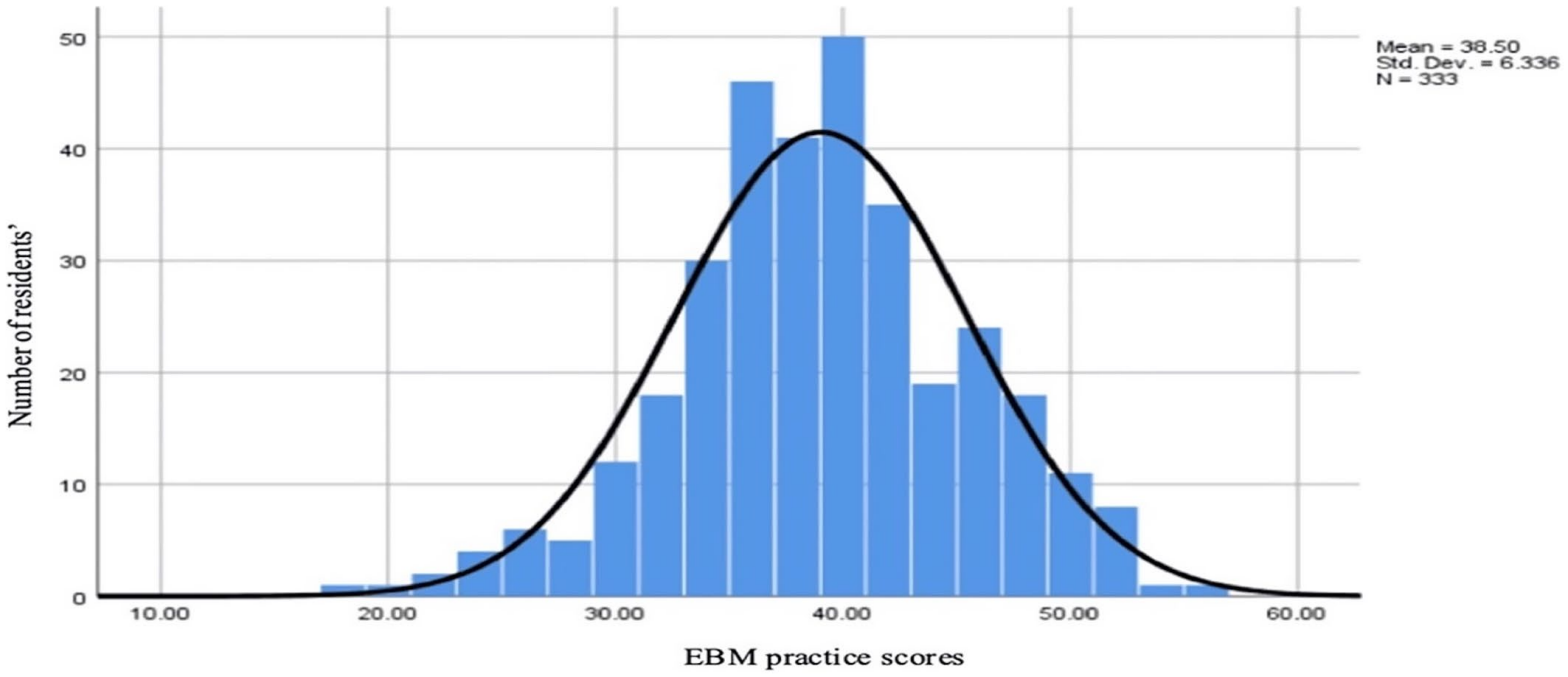
Distribution of total EBM practice scores among resident physicians in Qatar, 2024 (*N* = 355). The data have been presented as *N* and mean ± SD.

The mean EBM practice score among the residents was 38.49, and the majority (62.3%) were classified as having a moderate level of EBM practice according to Bloom’s classification. The distribution of these scores around the mean was considered normal, as shown in Figure 3.

As shown in Table 2, the mean knowledge score of EBM among residents was 51.6, with the majority (294; 83.4%) classified as moderate according to Bloom’s classification for the knowledge domain. The mean EBM attitude score among the residents was 67.24, and the majority of residents (202; 57.2%) scored low in Bloom’s classification for the attitude domain. However, the attitude scores showed minimal skewness, indicating a fairly symmetrical distribution.

The cutoff points of the 75th percentile were used to discriminate high self-efficacy from low self-efficacy. The mean EBM self-efficacy score among the residents was 28.64, with the majority of residents (287; 81.4%) having low EBM self-efficacy (Table 2).

### 3.3 Association between sociodemographic characteristics and EBM practice

**Figure 4:**
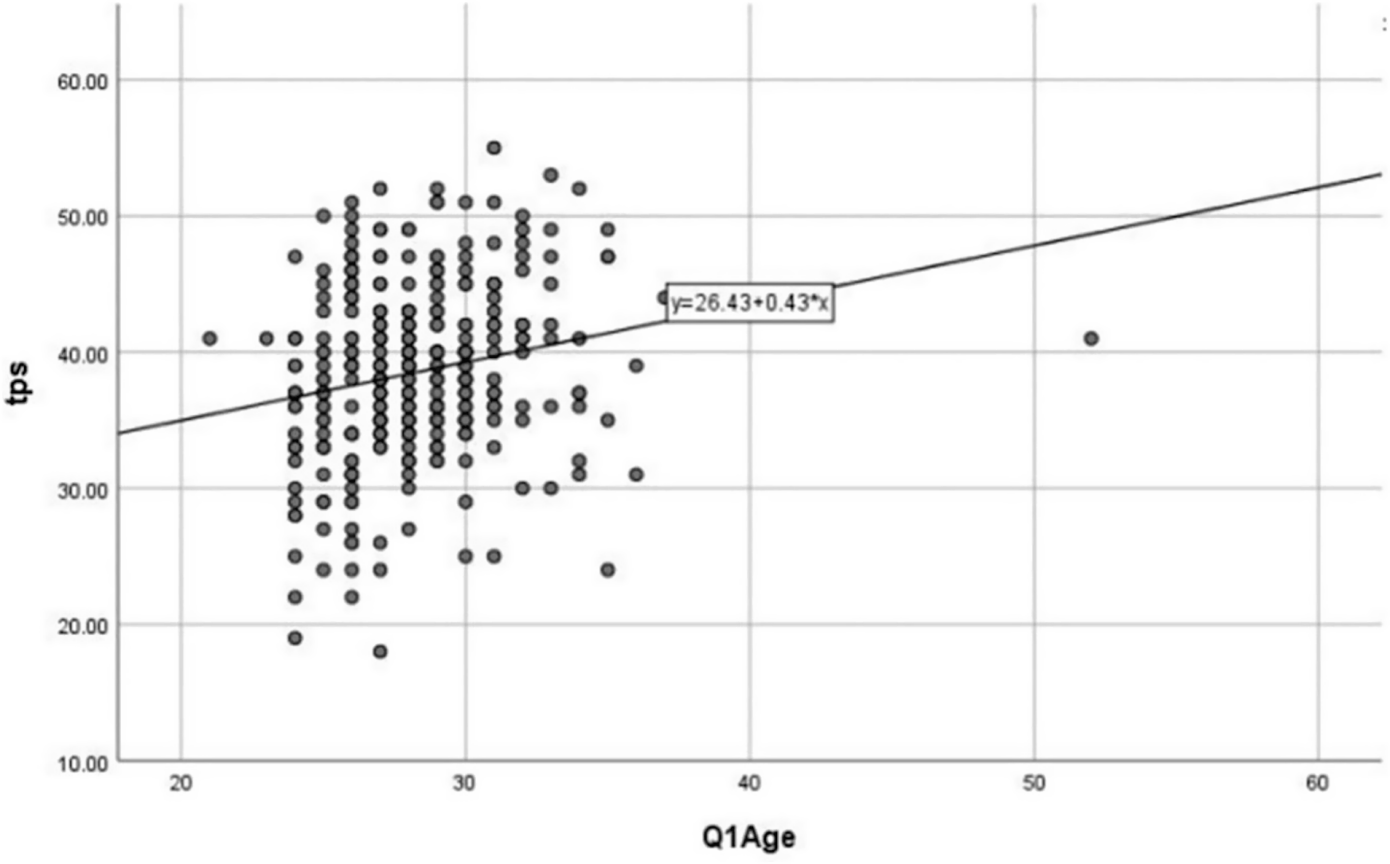
Correlation between age and total EBM practice scores among resident physicians in Qatar, 2024 (*N* = 355). The data have been presented as a scatter plot showing the relationship between age and EBM practice score, with an *R* value of 0.43, indicating a moderate positive correlation, statistically significant at a *p*-value of < 0.05.

As presented in Tables *3*, a statistically significant relationship was observed between resident physicians’ gender and nationality and their overall scores in the evidence-based medicine (EBM) practice domain. Additionally, a positive correlation was found between participants’ age and total EBM practice scores, which was statistically significant (*p* < 0.05), as illustrated in Figure 4.

**Table 3:** Association between residents’ sociodemographic characteristics and EBM-related practice, *N* = 355.

Variables	Frequency	Mean practice score	(SD)	*t*-test *t-*value	ANOVA test *F*-value	Correlation	*p*- value
Gender							
Male	180	39.23	−5.6	2.15	–	–	0.032*
Female	170	37.73	−7.02	2.13	–	–	–
Nationality							
Qatari	56	36.88	−7.57	−2.00	–	–	0.045*
Non-Qatari	283	38.79	−6.04	−1.72	–	–	–
Marital status							
Single	207	38.49	−6.58	–	0.02	–	0.973
Married	139	38.54	−6.01	–	–	–	–
Divorced	4	39.33	−8.38	–	–	–	–
Previous training in EBM							
Yes	152	37.47	−6.36	2.55	–	–	0.000*
No	179	39.28	−6.27	2.55	–	–	–
Type of training in EBM							
Combo	22	41.9	−6.35	–	3.45	–	0.000*
Face to face	117	39.04	−6.21	–	–	–	–
Online	30	16.32	−5.41	–	–	–	–
Previous research experience							
Yes	32	38.7	−6.48	1.76	–	–	0.201
No	316	36.56	−4.42	2.40	–	–	–
Specialty							
Internal medicine	103	28.90%	–	–	−0.25	–	0.082
Other	249	71.00%	–	–	−0.27	–	–
Time since graduation	–	4.47	2.56	–	–	0.18	0.000*
PGY level							
R1	92	25.80%	–	–	2.01	–	0.06
R2	105	29.50%	–	–	–	–	–
R3	69	19.40%	–	–	–	–	–
R4	61	17.10%	–	–	–	–	–

The data have been presented as *N*, % and mean ± SD, *p*-value (<0.05) is considered significant. *Indicate statistically significant.

Further analysis revealed that previous EBM training, time since graduation, and years of work experience were significantly associated with higher EBM practice scores. However, no significant relationship was identified between prior research experience and scores in the EBM practice score.

Workplace-related factors, including consistent Internet access, availability of medical databases, and participation in continuing medical education, did not show a statistically significant association with EBM practice scores among resident physicians.

No statistically significant associations were found between resident physicians’ gender, nationality, marital status, and their overall knowledge scores in evidence-based medicine (EBM). Additionally, no significant correlation (*p* > 0.05) was observed between age and EBM knowledge scores, as indicated by a linear correlation. However, a significant association was found between EBM training and higher total EBM knowledge scores. No significant relationship was found between prior research experience and EBM knowledge scores.

Similarly, workplace factors such as Internet availability, access to medical databases, and participation in continuous medical education did not show any statistically significant association with the EBM knowledge scores of resident physicians.

Regarding the attitude domain, no significant associations were observed between gender, nationality, marital status, and EBM attitude scores. Furthermore, no significant correlation (*p* > 0.05) was found between physician age and attitude scores, as demonstrated by a linear correlation. Nevertheless, a significant association was identified between prior EBM training and EBM attitude scores, while no significant relationship was noted between previous research experience and EBM attitude scores.

For self-efficacy in EBM, no significant associations were observed with gender, nationality, or marital status. However, a significant positive correlation was found between physician age and EBM self-efficacy, with a statistically significant (*p* < 0.05) relationship between age and total EBM self-efficacy scores among resident physicians. Additionally, prior EBM training was significantly associated with higher self-efficacy scores, whereas no significant relationship was found between prior research experience and EBM self-efficacy.

A significant positive correlation was observed between EBM knowledge and EBM practice, with a Pearson’s correlation coefficient of 0.202 and *p* < 0.000. Similarly, a statistically significant relationship was found between EBM attitude and EBM practice, with a Pearson’s correlation coefficient of 0.236 and *p* < 0.000. Furthermore, a strong positive correlation was identified between EBM self-efficacy and EBM practice, with a Pearson’s correlation coefficient of 0.620 and *p* < 0.000. Additionally, a notable relationship was found between EBM knowledge and EBM attitudes among resident physicians at Hamad Medical Corporation (HMC).

### 3.4 Predictors of EBM practice

The multivariate analysis revealed that several factors were significant predictors of EBM practice among resident physicians in Qatar in 2024. Specifically, older residents, those with higher EBM self-efficacy, and those displaying a positive attitude toward EBM were more likely to implement evidence-based practices, as shown in Table 4.

**Table 4:** Results of the linear regression to assess the predictors for evidence-based medicine practice.

Predictor	Adjusted analysis						Unadjusted analysis					
	*B*	SE	*β*	*T*	*p*	95.0% CI	*B*	SE	*β*	*T*	*p*	95.0% CI
Age	0.17	0.09	0.08	−0.26	0.05	−0.00 to 0.35	0.42	0.11	0.20	3.78	0.00	0.20–0.65
Gender	−0.41	0.56	−0.03	1.94	0.45	−1.52 to 0.69	−1.50	0.69	−0.11	−2.15	0.03	−2.87 to 0.13
Nationality (Qatari, non-Qatari)	1.04	0.77	0.06	−0.74	0.17	−0.47 to 2.27	1.91	0.95	0.11	2.00	0.04	0.04–3.78
Previous EBM training	−0.55	0.56	−0.06	1.35	0.32	−1.67 to 0.56	−1.80	0.70	−0.14	−2.55	0.01	−3.19 to 0.41
EBM knowledge	−0.00	0.06	−0.04	−0.98	0.88	−0.12 to 0.10	0.23	0.06	0.20	3.87	0.00	0.11–0.35
EBM attitude	0.09	0.04	0.10	−0.14	0.03	0.00–0.17	0.19	0.04	0.23	4.40	0.00	0.10–0.28
EBM self-efficacy	1.00	0.07	0.59	2.10	0.00	0.85–1.14	1.06	0.07	0.62	14.35	0.00	0.91–1.21
Constant	−1.22	4.59	–	–	0.79	−10.26-7.81	–	–	–	–	–	–

The data have been presented as unstandardized coefficients, *B*, unstandardized STD error, standardized coefficients beta, 95.0% confidence interval for *B*, and *P*-value (<0.05) is considered significant.

## 4 Discussion

In Qatar, where physicians come from a variety of medical backgrounds, it is crucial to establish a unified approach to medical practice. This study aimed to assess the adoption of evidence-based medicine (EBM) among resident physicians. Given that residents are early in their medical careers, they may be more open to integrating new practices into their clinical work. The research also explored the factors influencing EBM adoption among this group, providing valuable insights into their practice of medicine and how they incorporate evidence-based approaches into patient care.

The average age of participants was 28.3 years, which is consistent with the typical age range of medical residents (34). The male-to-female ratio of 1.1:1 (180:170) reflects the global trend of increasing female participation in medical education and residency programs (35). Participants had relatively limited work experience, with the majority having graduated from medical school within the past 5 years. The majority of the resident physicians received prior training in evidence-based medicine (EBM). Additionally, approximately 90% (320) of the residents had prior experience conducting research. The work environment at Hamad Medical Corporation (HMC), the main secondary care provider in Qatar, is highly supportive of EBM practice. Due to the country’s high-income status, residents have consistent Internet access at work, free access to medical databases, and regular opportunities for continuing education, advantages that may not be available in residency programs elsewhere.

The practice of evidence-based medicine (EBM) among the residents in this study was found to be moderate, which is better compared to EBM practice among primary care doctors in Selangor, emergency medicine residents in Malaysia (30, 36), and residents in Syria (15). A study conducted in Egypt reported that most resident physicians had poor EBM practice (14). Since all of these studies used the same validated questionnaire to assess EBM among residents, their results are comparable.

The highest mean practice score was achieved by Oral maxillofacial residents, and the lowest mean practice score was held by Radiology residents. In comparison to a study done in Syria using the same questionnaire, pediatric residents had the highest score in practicing EBM, while family medicine residents scored the lowest (15).

More than 87% (306) of participants in this study reported participating in Continuous Medical Education (CME) to stay updated on evidence-based medicine (EBM). This result is similar to studies conducted with resident physicians in Malaysia and primary care physicians in Selangor, where 90% and 94.2% of the respondents, respectively, used CME for EBM updates (30, 36). This high engagement with CME could be attributed to the Hamad Medical Corporation’s requirement for residents to participate in such educational activities to continue their training. In contrast, a study in Wuhan, China, found that only 61.8% of participants used CME as their primary source of information on EBM, compared to other channels such as school education, printed journals, the Internet, colleagues, and advanced training (37). Additionally, 38% (135) of our respondents selected “Sometimes” when asked if they lacked time to study EBM, which is a lower percentage than reported in Malaysia. In an Egyptian study, 60% of physicians identified lack of time as a significant barrier to practicing EBM (14). This finding aligns with other global studies, which also report time constraints as a barrier to EBM adoption (13, 38, 39).

The study also found that 99% (351) of respondents used multiple search engines for literature searches.

Similarly, over 90% of participants in the Malaysian study also reported using various search engines for this purpose. In our study, 67% (238) of participants frequently shared knowledge on EBM with colleagues in the workplace, a higher proportion than the 38% reported in Malaysia. The act of sharing EBM knowledge with colleagues is crucial, as highlighted by a study from Wuhan, which found that 34.4% of participants gained EBM insights through colleagues (37).

However, colleagues’ attitudes can be an obstacle to practicing EBM. This barrier was identified by Abdel-Kareem, who found that 47% of participants in his study saw their colleagues’ attitudes as a hindrance to EBM practice (14). A systematic review of EBM found that many physicians tend to consult their colleagues or field experts when faced with clinical questions (13). Common barriers to effective EBM practice identified in previous research include negative attitudes toward EBM, limited access to information, lack of time, and insufficient critical appraisal skills. In our study, gender, prior EBM training, high EBM knowledge, and neutral attitudes were found to be significantly associated with EBM practice. Other research has identified factors such as nationality, attitude, work experience, and access to online resources as key determinants of EBM practice (40). Interestingly, we found that female healthcare providers tended to practice EBM less effectively than their male counterparts. This may reflect patterns noted in previous literature, where female professionals often pursue less clinical roles to navigate gender-based barriers to clinical and administrative advancement (41). However, further research is needed to explore this finding in depth, as this difference is not widely studied in the existing literature.

Although this study did not find a significant association between medical specialty and EBM practice, it is possible that such an association exists but was not detected due to limited statistical power to assess specialty as a contributing factor to EBM practice.

Both time since graduation and prior research experience showed some correlation with EBM practice. A multivariate regression analysis indicated that high self-efficacy in EBM and a positive attitude toward EBM were the primary predictors of effective EBM practice among resident physicians.

The study also revealed that 83.7% (295) of residents had moderate EBM knowledge, while only 8% (28.4) demonstrated high knowledge. Factors such as prior EBM training were significantly linked to higher EBM knowledge levels. The highest knowledge scores were observed among urology and community medicine residents, followed by those in general surgery. In contrast, a study in Malaysia found that 49.7% of emergency medicine residents had high EBM knowledge, which is notably higher than the 8% (28.4) high knowledge rate in our study. This discrepancy may stem from the differing classification systems used to assess EBM knowledge in the two studies. In comparison, a survey of primary care physicians in Selangor, Malaysia, found that 60.9% had moderate EBM knowledge, while only 6.2% (22) exhibited low knowledge. Our study found a slightly lower level of EBM knowledge.

However, findings from other countries differ. For example, in Sri Lanka, less than 38% of medical officers understood complex statistical terms such as systematic reviews and meta-analyses (40). On the other hand, one-third of doctors in England were able to explain these terms (42). In our study, nearly all participants understood that EBM involves critically appraising research findings to guide clinical decisions, a finding consistent with a study on emergency medicine residents in Malaysia.

However, 44% (156) of respondents mistakenly believed that EBM focuses exclusively on the best available research, without considering clinical experience, similar to findings in the Malaysian study.

Regarding patient preferences in clinical decision-making, more than half of our respondents (54%; 192) incorrectly assumed that patient preferences were not an essential component of EBM, which mirrors the findings of the Malaysian emergency medicine resident study. Similarly, a study conducted in Sri Lanka reported that only 61% of participants recognized the importance of considering patient preferences in EBM (40). These results suggest a gap in awareness regarding patient values and expectations in clinical decision-making.

Furthermore, the majority of our respondents 87% (309) correctly identified that EBM includes four essential components structured around the PICO format, which aligns with findings from a Malaysian study. The PICO framework helps clinicians formulate effective clinical questions, leading to high-quality evidence and clinical decisions (43). Our respondents also demonstrated strong knowledge of the hierarchical nature of study designs, with 79% (280) correctly ranking meta-analyses as having a higher level of evidence than case-control studies. This is consistent with findings from emergency medicine residents in Malaysia, who also ranked meta-analyses higher than other study types.

Regarding the impact of EBM on clinical practice, 68% (241) of our respondents recognized that it promotes self-directed learning, which is notably higher than 18% of residents in Malaysia who shared this view. Self-directed learning is vital in the medical field, as it encourages lifelong learning and adaptation to evolving medical knowledge. The process of self-directed learning aligns closely with the PICO framework, making it an integral part of EBM (44). Additionally, 72% (256) of our respondents acknowledged the cost-effectiveness of applying EBM in healthcare, a finding consistent with the Malaysian study.

Regarding attitudes, 43% (153) of our participants demonstrated a moderate to high attitude toward EBM, which is slightly higher than the 40% of emergency medicine residents in Malaysia who exhibited a positive attitude. The oral and maxillofacial residents in our study displayed the most positive attitude toward EBM, followed by dermatology residents. A study in Wuhan, China, found associations between medical specialties and EBM attitudes. Similarly, a study in Saudi Arabia reported varying EBM attitude scores among different specialties, with surgeons exhibiting the lowest scores and pediatricians the highest (45).

In conclusion, despite the challenges associated with EBM adoption, such as time constraints and colleagues’ attitudes, our study suggests that resident physicians at Hamad Medical Corporation possess a moderate understanding of EBM and have a generally positive outlook on its impact on patient outcomes. Efforts to improve EBM self-efficacy, especially among residents in specialized fields, could further enhance the implementation of evidence-based practices in clinical settings.

### 4.1 Limitations of the study

Despite its strengths, this study has several limitations. Resident physicians were often busy and had limited time to complete the questionnaires. The diverse backgrounds of our study population may have introduced non-response bias, selection bias, and social desirability bias. Additionally, the cross-sectional design of the study captures only a snapshot of EBM practices at a single point in time; this design may not fully reflect the evolving nature of medical residency. A longitudinal approach would provide more comprehensive insights into how residents’ practices and perceptions change during their training.

## 5 Conclusion

While most resident physicians at Hamad Medical Corporation in Qatar demonstrate moderate knowledge and practice of EBM, their attitudes and self-efficacy toward EBM are relatively low. Factors contributing to poor EBM practice include insufficient knowledge and the absence of prior training in EBM. The challenges and barriers identified in this study can serve as a foundation for developing targeted interventions to enhance EBM practice among residents. For example, incorporating evidence-based medicine (EBM) training to enhance knowledge, along with mentorship to strengthen self-efficacy, into residency programs could help address existing gaps.

Given the supportive work environment highlighted in the study, EBM training and mentorship programs could be effectively delivered through online platforms. This approach may be more practical within Qatar’s well-resourced healthcare system and aligns well with its existing educational infrastructure.

Improving EBM practices will enable residents to better address local healthcare challenges, promote their professional growth, drive innovation, and ultimately enhance patient care quality.

## Data Availability

The raw data supporting the conclusions of this article will be made available by the authors, without undue reservation.
